# Strain induced piezoelectric effect in black phosphorus and MoS_2_ van der Waals heterostructure

**DOI:** 10.1038/srep16448

**Published:** 2015-11-10

**Authors:** Le Huang, Yan Li, Zhongming Wei, Jingbo Li

**Affiliations:** 1 State Key Laboratory for Superlattice and Microstructures, Institute of Semiconductors, Chinese Academy of Sciences, Beijing 100083, China

## Abstract

The structural, electronic, transport and optical properties of black phosphorus/MoS_2_ (BP/MoS_2_) van der Waals (vdw) heterostructure are investigated by using first principles calculations. The band gap of BP/MoS_2_ bilayer decreases with the applied normal compressive strain and a semiconductor-to-metal transition is observed when the applied strain is more than 0.85 Å. BP/MoS_2_ bilayer also exhibits modulation of its carrier effective mass and carrier concentration by the applied compressive strain, suggesting that mobility engineering and good piezoelectric effect can be realized in BP/MoS_2_ heterostructure. Because the type-II band alignment can facilitate the separation of photo-excited electrons and holes, and it can benefit from the great absorption coefficient in ultra-violet region, the BP/MoS_2_ shows great potential to be a very efficient ultra-violet photodetector.

Despite being a very promising two-dimensional (2D) material, gapless graphene has limitation in its applications in nanoelectronics and optoelectronics^1^,^2^,^3^. As alternatives, new researches have emerged focusing on other 2D materials such as transition metal sulfides (TMDs), which possess sizable band gap and display advantageous optoelectronic properties^4^,^5^,^6^,^7^. For example, bulk MoX_2_ (X = S, Se, Te) and WX_2_ are indirect band gap semiconductors, whereas their monolayers have direct band gaps, which are favorable for optoelectronic applications. The single-layer MoS_2_ based field-effect transistors exhibit an excellent current on/off ratio of 10^8 ^8, and the application of monolayer MoS_2_ in integrated circuits and logic operations has already been realized^9^.

Recently, a new 2D layered material, namely black phosphorus (BP) has been fabricated by several research groups^10^,^11^,^12^. Similar to other 2D materials, different BP layers binding together via the weak van der Waals (vdW) force. Theoretical calculations show that the band gap of BP is layer dependent with 0.30 eV in bulk BP and 0.90 eV in its monolayer form^12^,^13^,^14^,^15^. What’s more, bulk BP shows a high hole mobility up to 10000 *cm*^2^/*Vs*^16^. And well-behaved p-type field-effect transistors with mobilities of up to 1000 *cm*^2^/*Vs* have been demonstrated on few-layer BP^10^. These properties make BP a potential candidate for novel applications in nanoelectronics and optoelectronics.

Recent studies have shown that hybrid systems consisting of various 2D materials would provide more opportunities for achieving desired electronic and optoelectronic properties^17^,^18^,^19^,^20^,^21^. For example, remarkable multiple optoelectronic functionality, including highly sensitive photodetection and gate-tunable persistent photoconductivity has been observed in the graphene/MoS_2_ vdW heterostructures^22^. The vertical field-effect transistors based on the graphene-WS_2_ heterostructures are also fabricated with unprecedented current modulation exceeding 10^6^ at room temperature^23^. Moreover, Yexin Deng *etc* demonstrated that a gate-tunable p-n diode based on a p-type BP/n-type monolayer MoS_2_ vdw p-n heterojunction shows a maximum photodetection responsivity of 418 mA/W at the wavelength of 633 *nm*^24^. An anomalous photoluminescence quenching is observed in artificial heterostacks of monolayer TMDs and few-layer BP^25^. J. E. Padilha *etc* reported that the Schottky barrier height and doping of phosphorus can be controlled by applying an external perpendicular electric field^26^.

In this work, we investigate the effect of normal compressive strain on the structural, electronic, transport and optical properties of semiconducting BP/MoS_2_ vdW heterostructure. The band gap of BP/MoS_2_ decreases with the applied strain and a semiconductor-to-metal transition is observed when the applied compressive strain is more than 0.85 Å. The compressive strain also exerts influence on the charge carrier effective mass and concentration, suggesting that mobility engineering and good piezoelectric effect can be realized in BP/MoS_2_ heterostructure. The calculated optical property of BP/MoS_2_ shows that BP/MoS_2_ may be a very efficient ultra-violet photodetector because the type-II band alignment can facilitate the separation of photo-excited electrons and holes, and it can benefit from the great absorption coefficient in ultra-violet region.

## Results and Discussion

**Figure 1:**
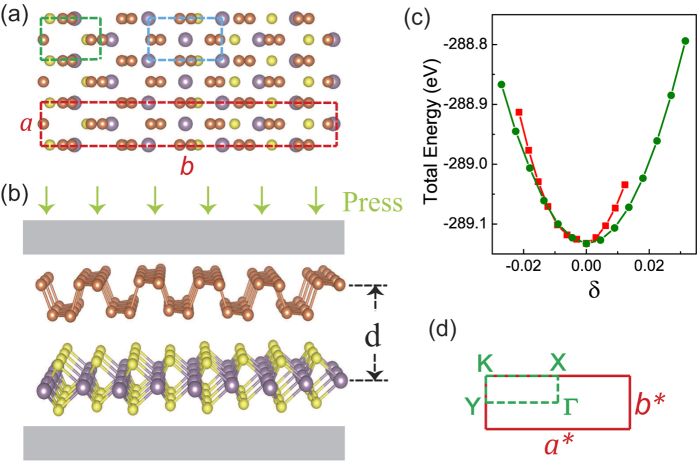
(**a**) Top view and (**b**) side view of BP/MoS_2_ bilayer. The green and blue rectangular regions present the unit cells of BP and MoS_2_. The supercell of BP/MoS_2_ bilayer is depicted in red rectangular region. (**c**) Evolution of the total energy of BP/MoS_2_ bilayer as a function of uniaxial strains. (**d**) Brillouin zone with high-symmetry points labeled.

Top and side views of the relaxed atomic structure of BP/MoS_2_ bilayer are shown in Fig. 1. To minimize the lattice mismatch between the stacking sheets, a rectangular unit cell of MoS_2_ is constructed. The supercell of this system is composed by 1 × 4 unit cells of BP and 1 × 5 unit cells of MoS_2_, which is the same with our previous work^27^. The optimised lattice constants of monolayer MoS_2_ are *a*_*M*_ = 3.19 Å, *b*_*M*_ = 5.53 Å and the calculated lattice constants of monolayer BP are *a*_*P*_ = 3.30 Å, *b*_*P*_ = 4.62 Å. To determine the stable structure of BP/MoS_2_ bilayer, the total energy as a function of uniaxial strains along X (zigzag) and Y (armchair) directions is depicted in Fig. 1(c). Both curves show their minimas under zero strain, at which point, the lattice constants of the supercell employed here are *a* = 3.26 Å, *b* = 22.18 Å.

**Figure 2:**
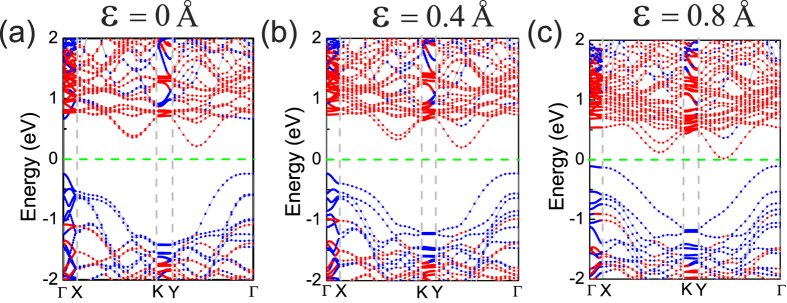
Evolution of projected band structure of BP/MoS_2_ bilayer as a function of the applied compressive strain. The bands dominated by BP and MoS_2_ are plotted by blue red dots, respectively.

Tuning the band gaps of 2D materials has been a challenge in band gap engineering. Many techniques, such as in-plane strains^28^,^29^,^30^,^31^ and a strong external electric field^32^,^33^, are promising but suffer from lack of practical applicability. Therefore, in this work, the effect of applied normal compressive strain on the electronic and optical properties of BP/MoS_2_ is studied. The strain is calculated as *ε* = *d*_0_ − *d*, where *d*_0_ and *d* are the equilibrium and instantaneous distances between the top phosphorus atom layer and the molybdenum atom layer. In Fig. 2, we show the projected band structure of BP/MoS_2_ bilayer under different compressive strain. Figure 1(d) presents the Brillouin zone of rectangular cells with high-symmetry points labeled. In Fig. 2, the bands dominated by BP and MoS_2_ are plotted by blue red dots, respectively. It is clear that the conduction band minimum (CBM) and the valence band maximum (VBM) are dominated by MoS_2_ and BP, respectively, regardless of the applied compressive strain. More importantly, BP/MoS_2_ vdW heterostructure has a type-II band alignment and thus the lowest energy electron-hole pairs are spatially separated with electrons and holes locating in MoS_2_ and BP layer, respectively. These results indicate that BP/MoS_2_ vdW heterostructure may be suitable for optoelectronics and solar energy conversion.

**Figure 3:**
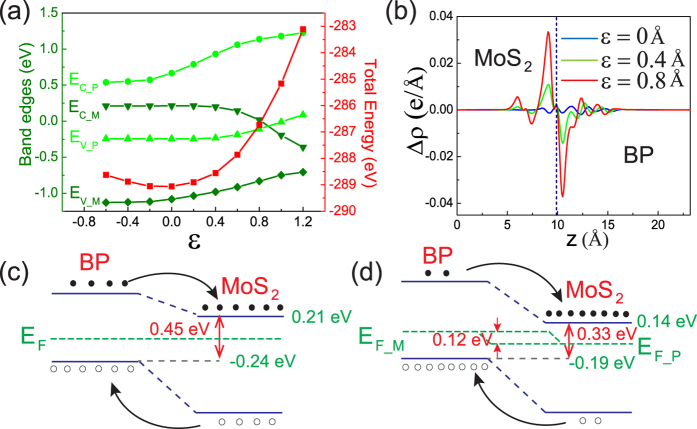
(**a**) Evolution of band edges and total energy of BP/MoS_2_ bilayer as a function of the applied compressive strain. (**b**) The difference between the integrated charge density of BP/MoS_2_ bilayer under different compressive strain and that of the isolated monolayers. (**c**,**d**) The band alignment of BP/MoS_2_ bilayer under applied compressive strain of 0 Å and 0.4 Å, respectively. *E*_*F*_*P*(*M*)_ is the quasi-fermi level of BP (MoS_2_) in BP/MoS_2_ bilayer.

Evolution of band edges and total energy of BP/MoS_2_ bilayer as a function of the applied compressive strain is concluded in Fig. 3(a). It can be proved that the interlayer distance *d*_0_ at equilibrium state is 6.92 Å. The vdW interaction exerts little influence on the band edges of BP/MoS_2_. The BP/MoS_2_ bilayer shows a finite indirect band gap up to 0.45 eV. Upon applying compressive strain, both the VBM and CBM move towards the Fermi level, resulting in a decreasing band gap with the applied strain. The BP/MoS_2_ bilayer experiences a semiconductor-to-metal transition when the applied compressive strain is larger than 0.85 Å, which may lead to tunable conductivity and transport properties.

The difference between the integrated charge density of BP/MoS_2_ bilayer under different compressive strain and that of the isolated monolayers as a function of the perpendicular distance is shown in Fig. 3(b). It is calculated as


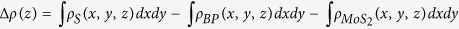


where *ρ*_*s*_(*x*, *y*, *z*), *ρ*_*BP*_(*x*, *y*, *z*) and 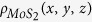 are the charge density at the (x, y, z) point in BP/MoS_2_ supercell, BP and MoS_2_ monolayer supercell, respectively. It is found that there is a small amount of charge exchange between BP layer to MoS_2_ layer. Furthermore, the applied compressive strain can facilitate electrons transferring from BP to MoS_2_ layer and holes transferring from MoS_2_ to BP layer. More charge transfer between BP layer and MoS_2_ layer suggests an increased carrier concentration and a stronger interlayer interaction. When under compressive strain, the quasi-fermi level of MoS_2_ moves upward and the quasi-fermi level of BP layer moves downward, as shown in Fig. 3(c,d). As a result, the band gap of BP/MoS_2_ bilayer decreases with the applied compressive strain.

**Figure 4:**
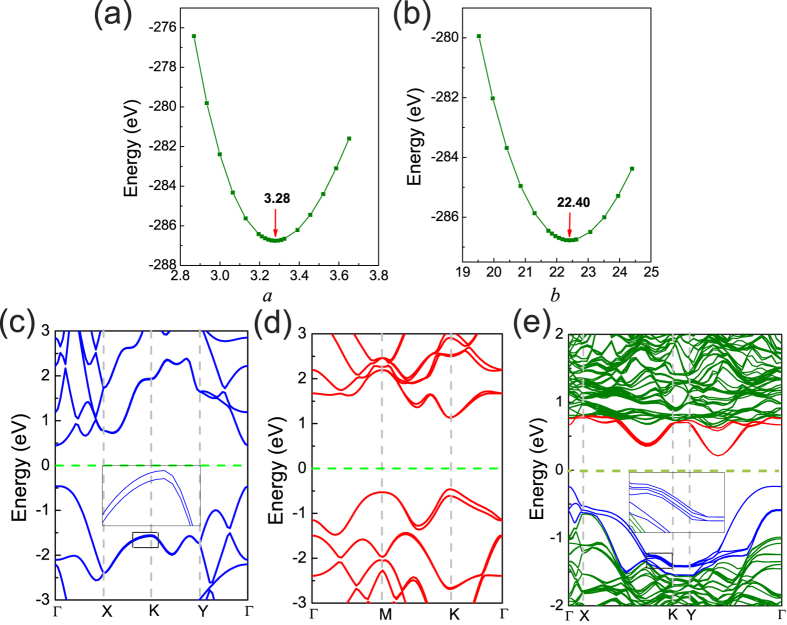
(**a**,**b**)The variation of the total energy with planar lattice constant, *a* and *b*, in BP/MoS_2_ bilayer under a normal compressive stran of 0.8 Å. The band structures of BP monolayer, MoS_2_ monolayer and BP/MoS_2_ heterostructure, including SOC effect are shown in (**c**–**e**) respectively.

For a three-dimensional material, when it is compressed along z direction, it usually will expand in the planar x and y directions. This effect is also taken into consideration in this work. Figure 4(a,b) gives the variation of the total energy with planar lattice constant, *a* and *b*, in BP/MoS_2_ bilayer under a normal compressive stran of 0.8 Å. It is found that the total energy reaches its minima when *a* = 3.28 Å, *b* = 22.40 Å, respectively. Both *a* and *b* are a little larger than that of BP/MoS_2_ bilayer without strains. It can be concluded that for the BP/MoS_2_ heterostructure, when exerting compressive strain along z direction, it also will expand in the planar x and y direction, just as a three-dimensional material.

Monolayer MoS_2_ has been reported to have a intrinsic spin-orbit gap. Whether the spin-orbit coupling (SOC) effect will exert obvious influence on the band gap of BP/MoS_2_ heterostructure is unknown. In Fig. 4(c,d), the band structures of BP and MoS_2_ monolayer, including SOC effect, are shown. BP and MoS_2_ monolayer show a spin-orbit gap of 22 meV and 147 meV at K point in the valence band, which are in good agreement with previous works^15^,^34^. As shown in Fig. 4(e), though the SOC gap of MoS_2_ can be comparable to the band gap of the BP/MoS_2_ heterostructure under compressive strain, the SOC exerts little influence on the band gap of BP/MoS_2_ because the VBM in the band structure of BP/MoS_2_ bilayer is dominated by BP whose SOC gap is very small rather than by MoS_2_. So the SOC effect is not taken into consideration in following results.

**Figure 5:**
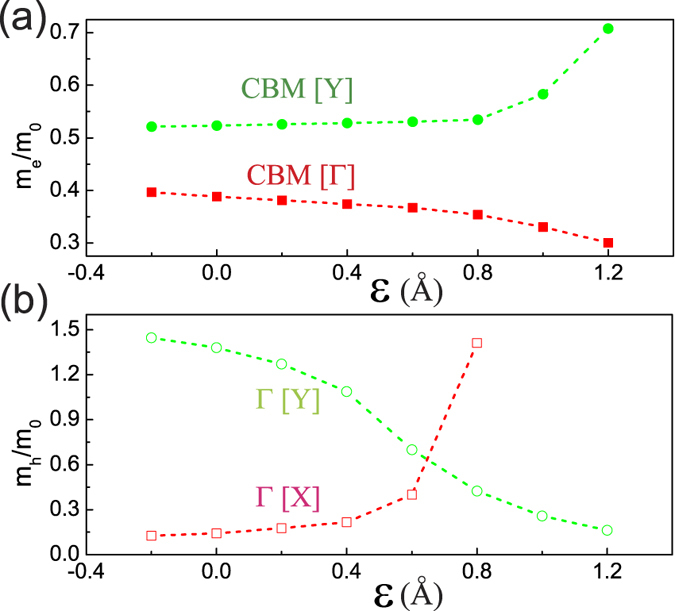
Effective masses (in units of electron mass m_0_) of (**a**) electrons and (**b**) holes as a function of applied compressive strain. The masses are labeled by the band extremum and the direction (in square brackets) along which the mass is calculated. The vertical dashed line indicates unstrained BP/MoS_2_.

With the applied normal compressive strain exerting influence on the band structure of BP/MoS_2_ bilayer, a change in the effective masses of electrons and holes can be expected. Figure 5 displays the changes of electron and hole effective masses with the compressive strain. The effective mass is calculated using 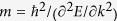, and the *k* points closely approach the VBM and CBM. In the band structure of BP/MoS_2_ bilayer, it is clear that the CBM is located at a point between Y and Γ. The curve labeled CBM[Y] shows the effective mass of electron at CBM along Y direction. The electron effective mass (*m*_*e*_) at CBM in the Γ direction is much smaller than that in the Y direction and it decreases gradually with applied compressive strain. The hole effective mass (*m*_*h*_) at Γ is quite sensitive to the compressive strain. *m*_*h*_ in the X direction increases drastically in the case of compressive strain larger than 0.4 Å, while that in the Y direction decreases gradually with the compressive strain. It also can be seen that the minimum of both *m*_*e*_ and *m*_*h*_ is much smaller than 1, suggesting that BP/MoS_2_ as a type-II heterostructure may possesses great transport properties such as high mobility and conductivity. BP/MoS_2_ vdW heterostructure may have great potential for applications in nanoelectronics and optoelectronics.

**Figure 6:**
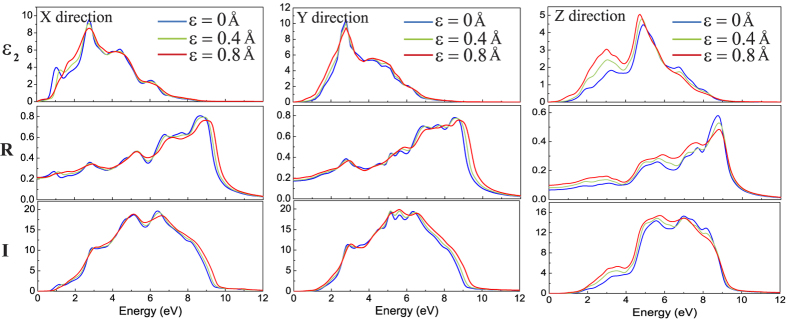
Calculated optical properties in (**a**) X directin, (**b**) Y direction and (**c**) perpendicular direction of BP/MoS_2_ bilayer under different compressive strain. From top to bottom planets are imaginary part of the dielectric function *ε*_2_, reflectivity *R* and absorption coefficient *I*, respectively.

Very recently, it is predicted that the BP/TMD system is a more efficient solar cell than the graphene/TMD systems^35^,^36^,^37^ because the former can benefit from the absorption of wider range of wavelength in the solar spectrum, and the type-II heterojunction alignment can allow more efficient hole-electron separation. In Fig. 6, therefore, the real (*ε*_1_) and imaginary (*ε*_2_) parts of the frequency-dependent dielectric functions are calculated by GGA-PBE and the frequency-dependent reflectivity *R* and absorption coefficient *I* are computed. Different with hexagonal lattice, the dielectric tensor has three independent components, namely, *ε*^*X*^, *ε*^*Y*^ and *ε*^⊥^. Strong anisotropy is observed in the imaginary parts of dielectric functions 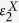, 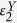 and 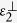. The variations of reflectivity and absorption coefficients as the function of frequency also exhibit similar trend, as illustrated in middle and bottom planets. From the absorption spectrum (the bottom planet), we can find that BP/MoS_2_ bilayer may have good application in ultraviolet light (above 3.27 eV) detecting. Furthermore, the effect of applied normal compressive strain on the optical properties of BP/MoS_2_ bilayer is also studied. The applied compressive strain shows little influence on the optical properties in the X and Y directions, while exerts much more remarkable influence on the three optical parameters in perpendicular direction (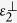, *R*^⊥^ and *I*^⊥^). It is because the compressive strain just enhances the interlayer interaction while exerts no influence on the in-plane interaction.

## Conclusion

In summary, we have provided total energy and band structure calculations for the p-type BP/n-type MoS_2_ vdW heterostructure and investigated its structural, electronic, transport and optical properties by using first principles calculations. The decreased band gap by applied compressive strain indicates the great application potential of BP/MoS_2_ vdW heterostructures in future nanoelectronics such as field effect transistors. A semiconductor-to-metal transition can be observed in BP/MoS_2_ bilayer under the compressive strain. The reduced carrier effective mass and improved carrier concentration by the applied compressive strain suggest that mobility engineering and good piezoelectric effect can be realized in BP/MoS_2_ bilayer. Furthermore, because the type-II heterojunction alignment can facilitate the separation of photo-excited electrons and holes, and it can benefit from the great absorption coefficient in ultra-violet region the BP/MoS_2_ heterostructure may be a very efficient ultra-violet photodetector. According to our results, the BP/MoS_2_ vdw p-n heterojuntion will present abundant opportunities for application in future nano- and optoelectronics such as photovoltaic cell, photodetector and logical device.

## Methods

The first-principles calculations are performed by VASP code within plane-wave basis sets and Perdew-Burke-Ernzerhof (PBE) projector augmented wave pseudopotentials^38^,^39^,^40^. The semi-empirical DFT-D2 method method of Grimme is utilized for the dispersion correction in the interlayer interaction^41^. The plane-wave cutoff energy is set to be 500 eV and a vacuum larger than 12 Å is used to simulate the isolated sheet. The first Brillouin zone is sampled with a (15 × 15 × 1) Monkhorst-Pack grid for relaxation of BP and MoS_2_ unitcells^42^. A (5 × 20 × 1) Monkhorst-Pack grid is used for relaxation of supercells. All the structures are fully relaxed with a force tolerance of 0.01 eV/Å.

## Additional Information

**How to cite this article**: Huang, L. *et al*.Strain induced piezoelectric effect in black phosphorus and MoS_2_ van der Waals heterostructure. *Sci. Rep*.**5**, 16448; doi: 10.1038/srep16448 (2015).
